# A high-cholesterol diet promotes the intravasation of breast tumor cells through an LDL–LDLR axis

**DOI:** 10.1038/s41598-024-59845-3

**Published:** 2024-04-24

**Authors:** Ana Magalhães, Vanessa Cesário, Diogo Coutinho, Inês Matias, Germana Domingues, Catarina Pinheiro, Teresa Serafim, Sérgio Dias

**Affiliations:** Instituto de Medicina Molecular João Lobo Antunes, Faculdade de Medicina, Universidade de Lisboa, Lisbon, Portugal

**Keywords:** Breast cancer, Mechanisms of disease

## Abstract

Most metastases in breast cancer occur via the dissemination of tumor cells through the bloodstream. How tumor cells enter the blood (intravasation) is, however, a poorly understood mechanism at the cellular and molecular levels. Particularly uncharacterized is how intravasation is affected by systemic nutrients. High levels of systemic LDL-cholesterol have been shown to contribute to breast cancer progression and metastasis in various models, but the cellular and molecular mechanisms involved are still undisclosed. Here we show that a high- cholesterol diet promotes intravasation in two mouse models of breast cancer and that this could be reverted by blocking LDL binding to LDLR in tumor cells. Moreover, we show that LDL promotes vascular invasion in vitro and the intercalation of tumor cells with endothelial cells, a phenotypic change resembling vascular mimicry (VM). At the molecular level, LDL increases the expression of SERPINE2, previously shown to be required for both VM and intravasation. Overall, our manuscript unravels novel mechanisms by which systemic hypercholesterolemia may affect the onset of metastatic breast cancer by favouring phenotypic changes in breast cancer cells and increasing intravasation.

## Introduction

Most deaths from breast cancer are due to metastases which may occur via lymphatic or hematogenous dissemination of tumor cells^1,2^. During hematogenous dissemination, tumor cells cross blood vessel endothelial beds at least twice, to enter circulation (intravasation) and exit from circulation (extravasation)^3^. These processes may be even more frequent, as re-seeding of primary sites, migration between metastatic sites and movement of disseminated tumor cells from the bone marrow to new metastatic sites, have been observed, both in clinical and pre-clinical studies^4–8^. Intravasation, however, is one of the least understood processes of the metastatic cascade^3,9,10^. It is believed that it can be an active or passive event and that it may be driven by tumor cell’s intrinsic and extrinsic factors, such as blood vessel permeability, macrophages and mechanical forces^11–13^. Additionally, both the intravasation of single cells and of oligoclusters of cells have been shown to occur^14^. In 2015, a study on breast cancer that used molecular barcoding to follow the path of metastatic cells from the primary to the metastatic site, suggested vascular mimicry (VM) as a driver of intravasation due to the action of two secreted proteins—SERPINE2 and Slpi^15^. How the expression of such molecules is induced and how their action allows cancer cells to do VM and intravasate remains to be elucidated. VM consists in the ability of tumor cells to form tube-like structures that allow blood flow through connection with the vascular system. It was first described in Human uveal melanoma^16^, but it was then observed in several other cancer types, including breast cancer and appears to associate with an aggressive disease^17^. While there are studies describing some molecular signatures involved in VM^18,19^, the cell biology behind this complex mechanism is vastly under-investigated. Nevertheless, VM and the establishment of mosaic vessels composed of both endothelial and cancer cells^11,20^, in tumors, include a set of cell and molecular changes which have been globally recognized as a novel hallmark of cancer (“unlocking phenotypic plasticity”)^21^.

Cholesterol is an integral component of cellular membranes where it affects membrane fluidity, trafficking and signaling through membrane receptors^22^. Although the generality of cells can synthesize cholesterol, the majority take it up from the plasma, through the internalization of the Low-Density Lipoprotein (LDL)^23^. LDL particles form in the blood from Very-Low-Density Lipoprotein (VLDL) that carries cholesterol, but mostly triglycerides. LDL is composed of cholesteryl esters, unesterified cholesterol, small amounts of triglycerides, phospholipids, and one single molecule of apoB100 protein. LDL enters cells mainly via the endocytic pathway through the binding of the apoB100 to the LDL receptor (LDLR). Following endocytosis, LDL is transported to the lysosome where it is degraded into unesterified cholesterol, free fatty acids, and free amino acids. Cholesterol is subsequently delivered to membranes or, as most mammalian cells are unable to catabolize cholesterol when in excess, is either secreted through ABC transporters into High-Density Lipoprotein (HDL) particles or stored in lipid droplets mainly as cholesteryl esters^24^. HDL is another plasma lipoprotein which role is to carry cholesterol back from tissues into the liver in order to maintain systemic cholesterol homeostasis. Plasma lipoprotein levels: LDL, HDL, and VLDL (triglycerides) are good indicators of the risk of cardiovascular diseases and, as such, are commonly assessed in the western population^25^. In cancer, particularly in breast cancer, several studies have looked for a possible relationship between plasma lipoprotein levels in the blood and cancer incidence, however, this has in general been difficult to ascertain^26^. In another hand, by looking at a possible association between systemic cholesterol and cancer progression and prognosis, we have previously shown that high systemic LDL at diagnosis, is associated with increased tumor size and aggressiveness and reduced disease-free survival in women with breast cancer^27^. This positive correlation between high systemic LDL and tumor progression is also supported by controlled mouse model studies in which either high-cholesterol diets or genetic models of hypercholesterolemia were used. Both strategies led to increased total circulating cholesterol and LDL and also to bigger and, in some cases, more metastatic tumors^28–32^. While some of the mechanisms through which high LDL may affect breast tumor size have been explored, it remains to understand how LDL levels may affect early events of the metastatic cascade such as intravasation.

Here we show that a high-cholesterol diet that dramatically raises LDL without affecting plasma triglyceride levels or body mass index (BMI)^33^, promotes intravasation of breast tumor cells in a way that is dependent on the internalization of LDL through the LDLR by tumor cells. Mechanistically, we suggest that the acquisition of a VM-like phenotype by breast cancer cells may be involved, as we were able to show in vitro that LDL promotes vascular invasion by tumor cells and the expression of SERPINE2.

## Results

**Figure 1:**
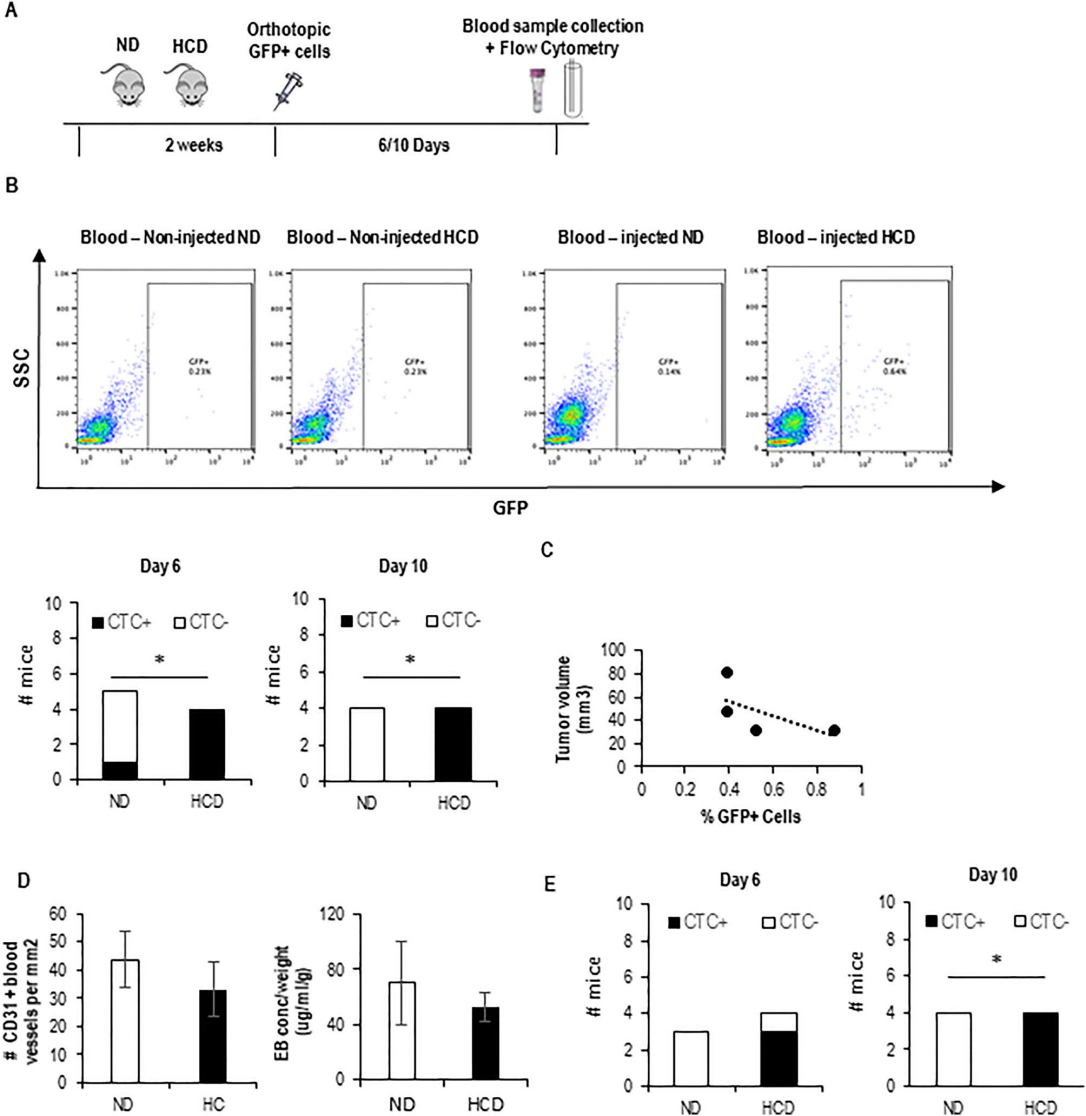
Short-term HCD potentiates the intravasation of BC cells. (**A**) Experimental design: mice were fed with either a normal diet (ND) or high cholesterol diet (HCD) for 2 weeks and then injected with GFP-expressing breast tumor cells at the mammary fat pad. Six and ten days after injection, blood samples were collected and analyzed by flow cytometry (FC) to search for the presence of GFP+ cells. (**B**) Representative plots for the FC analysis of blood samples from mice (BALB/c) that have not been injected with tumor cells (4T1): blood-non-injected (blank), and from blood samples from one representative mouse from each group that have been injected with tumor cells (injected ND and HCD). The bottom graphs show the number of mice in which CTCs were detected 6 days (n = 5 for ND and n = 4 for HCD) and 10 days (n = 4 for both groups) after injecting the tumor cells for each group. To statistically test for a relationship between the presence of CTCs in mice and the diet, a contingency table was used and a Fisher’s exact test applied (*p < 0.05). (**C**) On day 10, mice were sacrificed, tumors collected, and tumor size determined and plotted against the number of GFP+ tumor cells in circulation for each mouse. The graph shows data from HCD-fed mice, as only these mice had CTCs at day 10. (**D**) Number of CD31-expressing blood vessels (left) and permeability of blood vessels (right) were quantified for each mouse. Graphs shows means ± SD. (**E**) Same as in (**A**) and (**B**), but for the MDA-231 cell line on NSG mice (n = 3 ND, n = 4 HCD).

**Figure 2:**
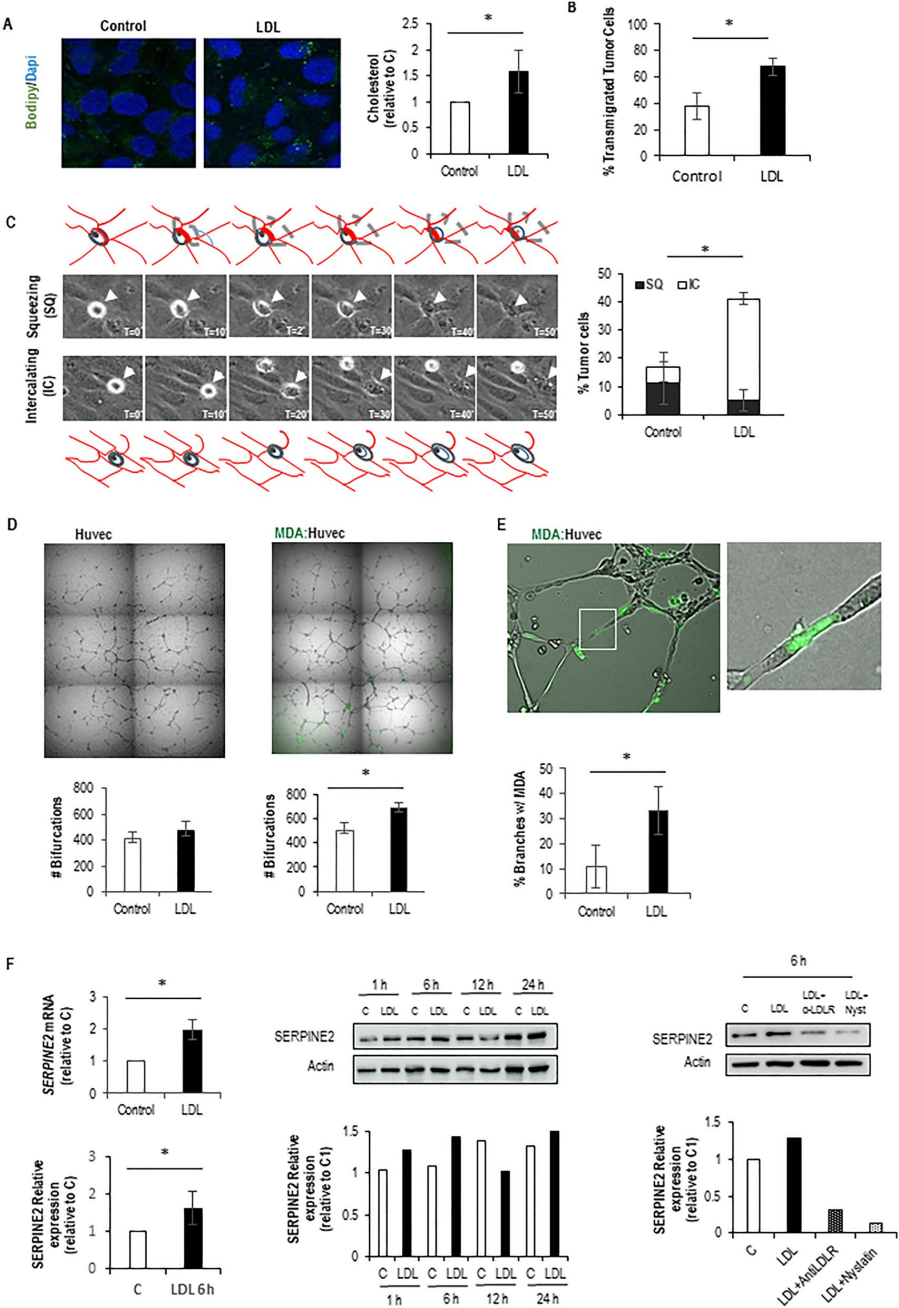
LDL uptake induces transendothelial migration and a vascular mimicry-like phenotype on tumor cells. (**A**) Lipid droplet staining with Bodipy (left) and cholesterol quantification (right) of tumor cells treated or untreated with LDL for 6 h. (**B**) Transendothelial migration on transwells of tumor cells pre-treated with LDL for 6 h. (**C**) Live imaging of tumor cells interacting with confluent endothelial monolayers in 2D, showing examples of tumor cells squeezing (SQ) or intercalating (IC) with endothelial cells (left) quantification of squeezing and intercalating events in control and LDL conditions (right). (**D**) 3D branch formation assay on Matrigel of endothelial cells only (left) and tumor cells co-cultured with endothelial cells in a 1:4 ratio (right), in either control or LDL conditions, and quantification of the number of branches for each condition (bottom panels). (**E**) Magnified picture of the tumor cell: endothelial cell co-culture presented on (**D**), to show a tumor cell sided by endothelial cells composing a branch and the quantification of the percentage of such structures in control versus LDL conditions. (**F**) QPCR (top Left) and western blot (bottom left) analysis of SERPINE2 mRNA and protein expression (respectively) upon exposure of tumor cells to LDL for 6 h as compared to control. Averages ± SD from three independent experiments are shown (*p < 0.05). Western blot analysis of SERPINE2 expression in tumor cells that have been exposed to either control, or LDL for 1, 6, 12 and 24 h (middle panel). Western blot analysis of SERPINE2 expression in tumor cells that have been exposed to either control, LDL and LDL + anti-LDLR antibodies and LDL + nystatin (Nyst) (right panel) this experiment has been repeated once (see Supp. Fig. 4). Original blots presented here in a cropped version are available in Supp. Fig. 4.

**Figure 3:**
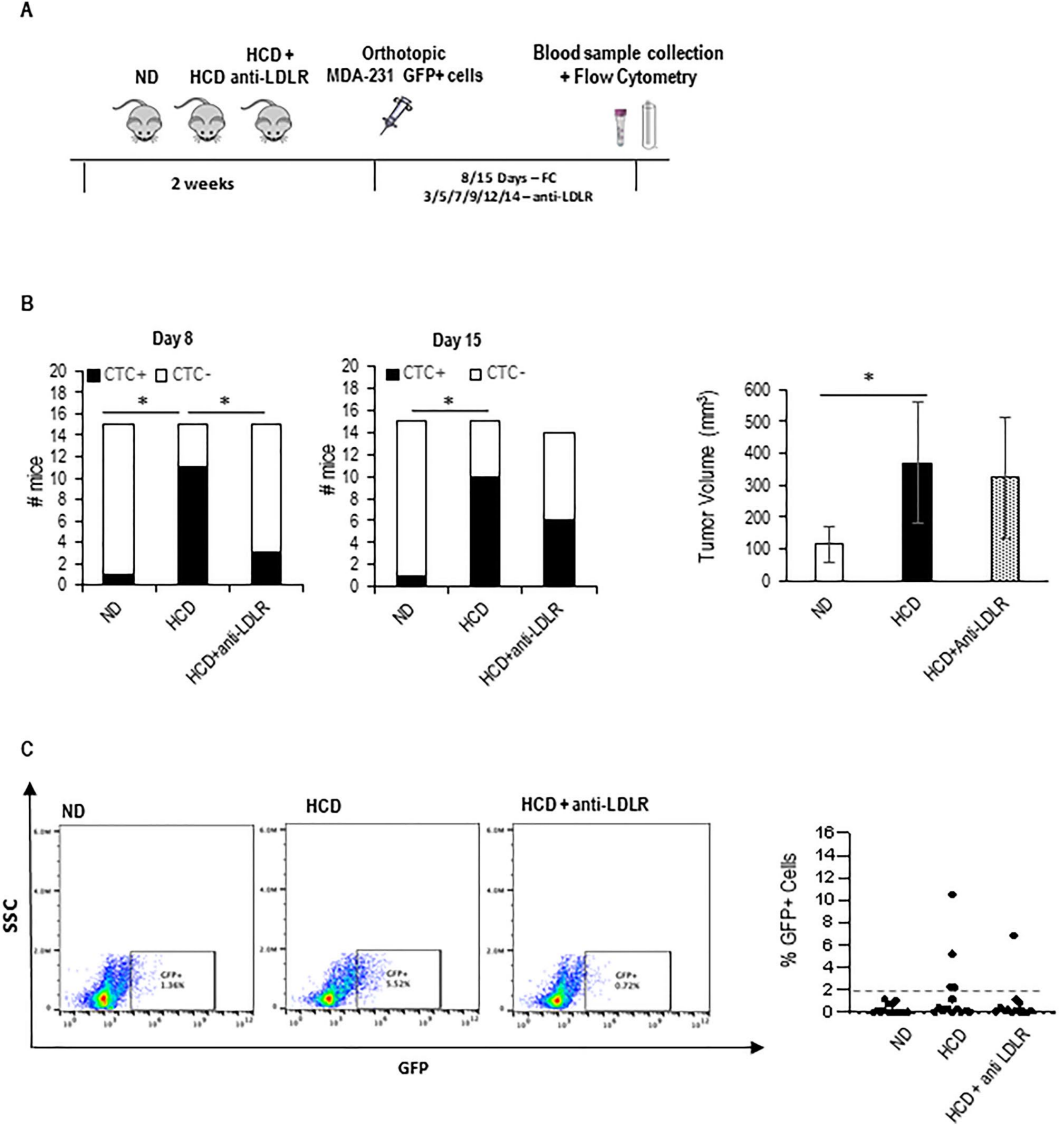
Tumor cell-specific LDLR blockage rescues HCD-induced intravasation and metastasis. (**A**) Experimental design: NSG mice were fed with either a normal diet (ND) or high cholesterol diet (HCD) for 2 weeks, and then injected with MDA-231 GFP+ breast tumor cells at the mammary fat pad. Cells were injected together with anti-LDLR or IgG control and then injected intra-mammary and IP every other day with the same antibodies. Eight and fifteen days after injection, blood samples were collected and analyzed by flow cytometry to search for the presence of GFP+ cells. (**B**) Number of mice with CTCs at days 8 and 15 after injection of tumor cells (Left and middle) (n = 15, except for HCD+ Anti-LDLR at day 15, when n = 14). On day sixteen mice were sacrificed, tumors collected, and tumor size determined and plotted against the number of GFP+ tumor cells in circulation for each mouse (right). (**C**) Lungs collected from sacrificed mice were digested and cells in suspension were taken to a flow cytometer to quantify GFP+  cells in the lungs. A representative dot plot of each group is shown (Left) and the average of the percentage (%) of GFP+ cells found in the lungs of each mouse is presented in the chart (n = 14 for ND and HCD, n = 13 HCD + IgG groups).

To address the question of whether a high-cholesterol diet affects the entry of breast tumor cells into the circulation, we injected GFP-expressing 4T1 cells into the mammary gland of BALB/c mice that were either fed on a normal diet or high cholesterol diet (HCD) for 2 weeks. We then looked for the presence of GFP+ circulating tumor cells (CTCs) in blood samples, over time, by flow cytometry (Fig. 1A). This approach allowed the detection of CTCs 6 and 10 days after injection of cells, almost exclusively in the HCD-fed mice (Fig. 1B, and Supp. Fig. 1). Interestingly the number of CTCs detected at day 10 does not positively correlate with tumor size or with increased blood vessel number and permeability (Fig. 1C,D). Similar results (CTCs detected on HCD-fed mice) were obtained with GFP-expressing MDA-231 cells injected into NSG mice (Fig. 1E and Supp. Fig. 2). These data suggest that a high-cholesterol diet affects the entry of tumor cells into circulation. As we have previously shown that the hypercholesterolemic feeding scheme used here dramatically increases systemic LDL levels^33^, we decided to test the hypothesis that LDL promotes tumor cell invasion of blood vessels. For that, we performed a transendothelial migration assay (in a transwell system) in which tumor cells were plated either in control or high LDL conditions for 6 h and then plated on top of endothelial cell monolayer. As a readout for LDL uptake, we visualized lipid droplets using BODIPY and measured intracellular cholesterol biochemically. As shown in Fig. 2A, a 6-h treatment of MDA-231 cells with LDL lead to an increase in lipid droplets and cholesterol content. Moreover, LDL-treated cells showed an increased capacity to transmigrate through endothelial monolayers (Fig. 2B). To gain further insights into how tumor cells and endothelial cells interact with each other in high LDL conditions we performed time-lapse imaging of either control or LDL-treated tumor cells approaching endothelial monolayers. This allowed the distinction of two ways by which tumor cells interact with endothelial cells in a monolayer: tumor cells either squeeze between two endothelial cells and place themselves underneath the endothelial cells (Fig. 2C—top panel and movie 1a—tumor cell on the left), or they intercalate with endothelial cells and become part of the monolayer in a process we named as intercalation (Fig. 2C—bottom panel, movie 1a—tumor cell on the right and movie 1b and Supplementary Fig. 3A). The quantification of both events separately revealed that high LDL was mainly affecting the intercalation event—potentiating it. Given the resemblance of the intercalation event to VM/mosaic vessel formation, which have been previously linked to intravasation^11,15,20^, we conducted an in vitro assay to simulate VM/mosaic vessels under both control or high LDL conditions. For this, endothelial cells and GFP+ tumor cells were placed on top of Matrigel in a ratio of 1:4, and 16 h later, cells were observed using a fluorescent microscope (Fig. 2D,E). We were able to see that tumor cells intercalate with endothelial cells, also in this setting, and integrate the branches that endothelial cells typically form in such culture conditions (Fig. 2D—right and Fig. 2E, movie 2 and Supp. Fig. 3B). Interestingly, the number of branches containing intercalated tumor cells was higher in high LDL conditions as compared to control conditions (Fig. 2E). The number of bifurcations was also increased in the presence of LDL in the co-culture system (Fig. 2D—right), while LDL treatment did not affect the number of bifurcations formed by endothelial cells when plated alone (Fig. 2D—left). Together, these data suggest that LDL potentiates the intercalation of cancer cells with endothelial cells in a VM/mosaic-vessel-like behavior. Since VM has been previously suggested to promote intravasation in a way that was dependent on the action of SLPi and SERPINE2^15^, we decided to test whether LDL could affect the expression of these molecules. While no differences were detected in SLPi expression on high LDL as compared to control conditions, high LDL increased the expression of SERPINE2 both at the mRNA and at the protein level (Fig. 2F—left and middle panel and Supp. Fig. 4). Moreover, the effect of LDL on SERPINE2 expression was reverted by blocking the entry of LDL into tumor cells, using an anti-LDLR antibody, and sequestering cholesterol using nystatin (Fig. 2F—right panel and Supp. Fig. 4). Overall, our in vitro data suggest that high LDL potentiates the ability of tumor cells to intercalate and transmigrate endothelial cell monolayers in a way that resembles VM/mosaic vessel formation and may involve the increased expression of SERPINE2. As LDL-mediated increase in SERPINE2 expression was reverted by using an anti-Human LDLR receptor antibody, we then tested the hypothesis that this antibody could prevent the increased intravasation capacity of breast cancer cells mediated by the high cholesterol diet in vivo. For that, the NSG-MDA-231 system was instrumental. In support of our hypothesis, we observed that anti-LDLR partially reverted the effect of high cholesterol diet at promoting breast tumor cell’s intravasation in mice (Fig. 3A,B and Supp. Fig. 5), this was however, more evident at the earliest time point. Also interestingly, injections with anti-LDLR did not prevent the increase in tumor size mediated by the HCD (Fig. 3B—right panel).

Finally, we looked for the presence of disseminated tumor cells (DTCs) in the lungs of each mouse. We were able to observe that all mice, except 2 on ND, showed some dissemination of cancer cells in the lungs (Fig. 3C and Supp. Fig. 6). We also noticed that while in the ND-fed mice all mice showed very similar percentages of DTCs in the lungs, always below 2%, in the HCD-diet fed mice 35% of mice (3/9) showed a much higher percentage of DTCs (above 2%). In the case of the mice on HCD and treated with anti-LDLR only 8% (1/9) of mice have percentage of DTCs in the lungs higher that 2% (Fig. 3C and Supp. Fig. 6). These data suggest that the increase in CTC numbers and DTCs at the lungs observed in mice fed on HCD is dependent on the uptake of LDL by tumor cells through the LDLR. Importantly, we were not able to detect differences in the metastatic capacity of tumor cells that were injected in the tail-vein of mice that had been previously on an HCD as compared to mice fed on a normal diet (Supp. Fig. 7). This further suggests that the increase in lung metastasis promoted by the HCD is linked to the increased intravasation of breast cancer cells in the hypercholesterolemic environment.

**Figure 4:**
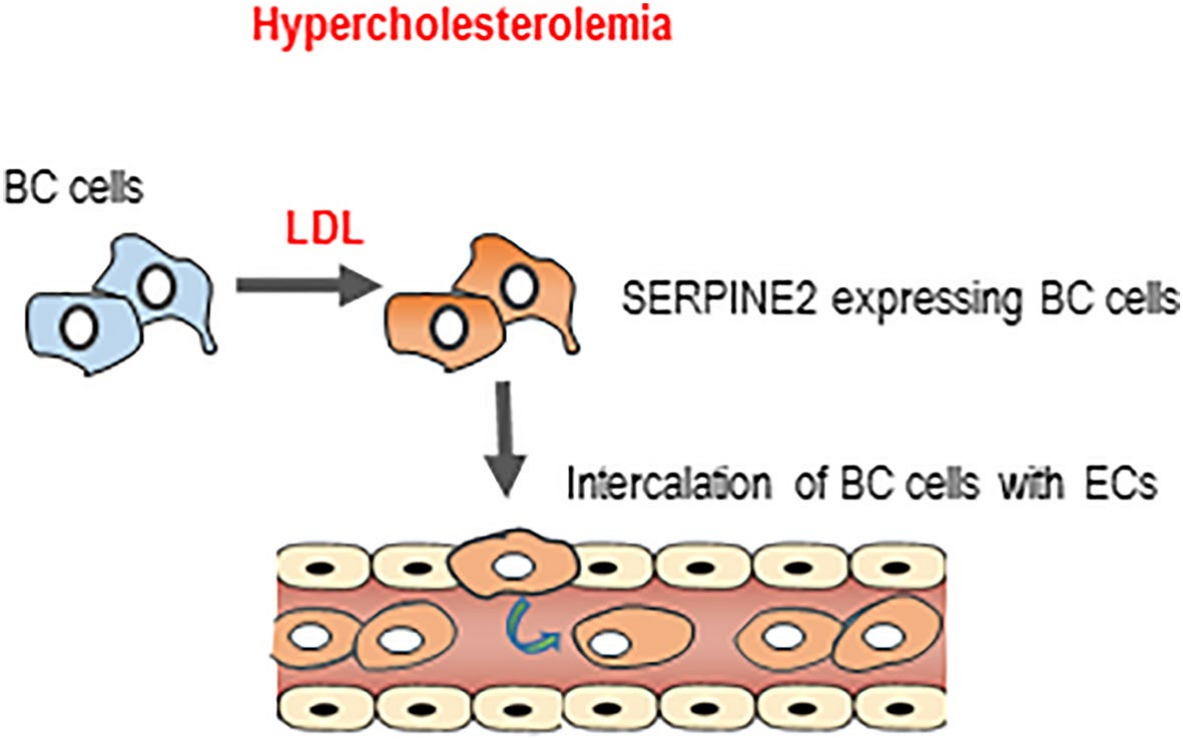
Working model. The data presented in this manuscript support the conclusion that in high systemic LDL conditions induced by a high cholesterol diet, breast tumor cells when exposed to high levels of LDL have increased intravasation capacity. Mechanistically, the data suggest that LDL induces the expression of SERPINE2 and the intercalation of tumor cells with endothelial cells. Tumor cell intercalation in blood vessels facilitates intravasation and increase the probability of metastasis.

Collectively, the findings presented in this study underscore the involvement of systemic metabolism in the aggressive and metastatic phenotypes of tumor cells (graphical representation of the model in Fig. 4). These results provide additional evidence for the detrimental effects of high systemic cholesterol environments in the context of breast cancer metastasis. Overall, it goes in line with the importance of controlling systemic LDL levels to help preventing breast cancer progression.

## Discussion

We have previously shown that a short-term high cholesterol-feeding scheme that raises systemic LDL levels promotes lung metastasis in mouse models^33^, here we searched for cellular and molecular mechanisms that could be responsible for that.

By using GFP-expressing breast tumor cells and blood sampling at different time points we were able to see that an HCD increases the numbers of CTCs detected in mice, suggesting it could affect intravasation. In previous papers that addressed the role of HCD in metastasis formation, the possibility that it could affect intravasation has not been addressed^28–32^. Several cellular processes enable tumor cells to enter circulation, ranging from passive to active mechanisms^9^. Considering the active mechanisms transendothelial migration, VM, and mosaic vessel formation have been pointed to as drivers of intravasation^11,15^. Our in vitro studies suggest that LDL is potentiating intravasation through a VM-like mechanism, as LDL promotes the intercalation of tumor cells with endothelial cells in both 2D and 3D cell culture conditions and the expression of SERPINE2. We have previously shown in a microarray study that treatment of MDA-231 with LDL induces the upregulation of several genes linked to cholesterol metabolism and also different cellular processes^33^. Among these were some SERPINS including *SERPINE2*. Here we confirmed this induction of *SERPINE2* expression through LDL by qPCR, and at the protein level by western blotting. Others have shown, in a different context, that LDL induces the expression of *SERPINE2* in vascular smooth muscle cells (vSMCs)^34^ and more recently in macrophages^35^. Here, we took a step further and showed that LDL-induced increase in *SERPINE2* levels in breast tumor cells is dependent on the internalization of LDL through the LDLR and on cholesterol itself. Requiring further investigation is the precise mechanism by which LDL induces the expression of SERPINE2 and how SERPINE2 promotes VM. Serpins are serine proteases that play important roles during coagulation, fibrinolysis and tissue remodeling. SERPINE2 has been shown to inhibit proteases such as trypsin, thrombin, and uPA and tPA, interestingly, it forms covalent complexes to its targets, which are cleared by endocytosis mainly via the LDL receptor-related protein (LRP1)^36,37^, a receptor that also internalizes LDL^38^. Whether this is linked to the effect of LDL on SERPINE2 expression requires, however, further investigation.

Our data suggest that one mechanism by which an HCD potentiates metastasis is by promoting intravasation through LDL acting directly on tumor cells, as LDLR blockage leads to a partial reversion of the HCD-induced intravasation and presence of DTCs in the lungs (represented graphically in Fig. 4). This model is also supported by the fact that we did not see differences in either the number or size of the metastatic foci in tail-vein injection experiments. This was true for both the 4T1-Balc/C model and the MDA-231-NSG model. These results may seem contradictory to data published by other groups who have shown that long-term high-fat diets leading to obesity contribute to increased colonization of the lungs upon tail-vein injection^39–41^. We believe, however, differences may relate to the composition of the diets and the extension of the feeding scheme: our diet has extra cholesterol and cholate and our feeding scheme is short and does not induce obesity^33^. Interestingly, our data also suggest the existence of a negative correlation between tumor size and CTCs in the high cholesterol setting (Fig. 1C), also 15 days after the injection of cancer cells, the effect of the HCD on CTCs is less pronounced as compared to earlier time points (Fig. 3B), this is surprising and may require further investigation, one possibility is that the HCD is affecting the intravasation capacity of a particular sub-population of tumor cells that is more present in the tumor when tumors are still small.

The metastatic cascade consists of a series of events and, most likely, many of such may be affected by high systemic cholesterol in circulation, here we are just identifying one of such mechanisms. In addition, for both CTC detection and lung metastasis we were only able to partially revert the effect of HCD using the anti-human LDLR, which suggests that other mechanisms must be occurring. These may range from the action of hypercholesterolemia of cells of the tumor microenvironment and on LDL acting to other LDL receptors, such as, for example CD36, which role in metastasis has already been described^42^. A cell type that is well known to be particularly affected by high systemic cholesterol levels are endothelial cells. We did not however detect major differences in the permeability of endothelial blood vessels at the tumor or increased extravasation of tumor cells at the lungs in mice that are fed on HCD as compared to mice on ND. This led us to follow a tumor-cell-centric mechanism to explain our data, however an effect of HCD on endothelial cells that would potentiate intravasation could not be excluded.

Our in vitro and in vivo data also suggest that the mechanisms governing the crossing of endothelial monolayers during intravasation and extravasation by cancer cells is most likely regulated by different mechanisms. We hypothesize that during extravasation, cancer cells squeeze their membrane in between endothelial cells and the binding of integrins to the endothelial basement membrane will generate the force that is required for the cell to go through the endothelial monolayer. During intravasation, as there is no physical support on the other side, cancer cells instead of squeezing trough endothelial cells, integrate the monolayer, maybe with forces being exerted towards the lateral sides and not basal-apically. We speculate that blood flow shear stress will then be the force that will lead to tumor cell release from the endothelial monolayer. In accordance to our hypothesis, others have shown the existence of a correlation between a vascular mimicry phenotype and intravasation^15^. Tumor cell exposure to high levels of LDL seems to be affecting the second, but not the first scenario.

Overall, our findings support the idea that LDL acts as a systemic factor capable of affecting cancer cell phenotypic plasticity. It identifies LDL as a trigger of intravasation in a hypercholesterolemia setting, while also showing that blocking the interaction of LDL with the LDLR partially reverts it. In the future, it would be interesting to address whether tumor cell populations with higher levels of LDLR or other LDL receptors would have higher intravasation capacity in a non-hypercholesterolemic situation. In human breast tumor samples, others have shown by looking at large data from international cohorts that LDLR expression is associated with a worse prognosis in patients who undergo systemic therapy^43^.

## Methods

### Animal work

Animals (10–12 weeks of age) were obtained from The Jackson Laboratory and all experiments were performed in accordance with the ARRIVE guidelines and Animal Care Committee of the Instituto de Medicina Molecular (iMM)—ORBEA, and approved by the competent Portuguese authority—Direcção-Geral de Alimentação e Veterinária (DGAV) following European Union guidelines. For all experiments, mice were fed either a normal diet (Placebo) or a special diet containing 15.8% Fat (coconut oil) + 1.25% Cholesterol, + 0.5% Na-Cholate, both from Ssniff and sacrificed at the end of each experiment through CO_2_ inhalation. Mouse wellbeing was closely monitored over the course of the experiment and mice were sacrificed if critical limits were achieved. Whenever this happened animals and datapoints had to be excluded from the analysis. Data exclusion also took place whenever enough blood collection wasn’t achieved due to technical reasons.

### Orthotopic injections

For orthotopic injections 1 × 10^6^ 4T1 cells expressing the green fluorescent protein (GFP) or 5 × 10^5^ MDA-MB-231 cells expressing GFP, were injected at the left fourth mammary fat pad (orthotopic injection). For the LDR blockage experiment, 3 µg of anti-LDLR antibody (R&D Systems, AF 2148) or IgG control, were injected together with tumor cells at day 0 and then intramammary and intraperitoneally every other day with the same amount of antibodies. At dissection, tumors and lungs were collected. Tumors were measured as length (L) and width (W), and tumor volume (TV) was calculated according to the following formula (L × W^2^)/2. Tumors were then collected into media, followed by gelatin embedding, finally frozen, and prepared for histological analysis at iMM’s histopathology facility.

### CTC detection

For the detection of CTCs, 50 µl of blood were collected via puncture of the submandibular vein with a lancet. Blood was then lysed with Red Blood Cell Lysis Buffer (Invitrogen) for 15 min, washed twice in PBS followed by 5 min centrifugation at 200*g* and finally taken to the flow cytometer—BD Accuri C6, and analyzed for the presence of GFP positive cells. Blood collected from normal diet and high-cholesterol diet-fed mice that were not injected with tumor cells were used as blanks and the % of cells FITC+ found in these samples subtracted from each mouse of the respective diet.

### Lung metastasis by flow cytometry

For detection of GFP-expressing cancer cells disseminated at the lungs after orthotopic injection, lungs were minced and then digested using 1 mg/ml collagenase type1A (Sigma) in media without FBS at 30 °C for 30 min. Digested lungs were then passed through a 70 µm nylon mesh strainer for cell isolation. Cells in solution were then washed once with PBS and red blood cells lysed with Red Blood Cell Lysis Buffer (Invitrogen) for 15 min. The obtained solution was finally centrifuged, washed once with PBS and then analyzed for the presence of GFP+ cells by flow Cytometry. Lungs from a mouse that was not injected with GFP-expressing tumor cells were used as blank.

### Evans blue permeability assay

Mice were injected intravenously (iv) with 0.2 ml of 1–2% Evans Blue (Sigma). Mice were sacrificed 1 h later, and tumors were weighed and placed in formamide (2 ml) (Merck) (37 °C, 48 h) to extract Evans Blue dye from the tissue. Absorbance was measured at λ = 620 nm (Bio-Rad SmartSpec 3000). Evans Blue concentration was calculated from a standard curve and is expressed as µg of Evans Blue per g of tumor tissue.

### Tail-vein injection

For tail-vein injections cells in culture were trypsinized and washed twice with PBS, before being resuspended in PBS. 1 × 10^6^ were injected in the tail vein of each mouse that were previously separated in two groups—normal diet-fed mice and high-cholesterol diet-fed mice for 2 weeks ad libitum. Mice were kept on the same diet regimen for another 2 weeks before being sacrificed and the lungs collected for histopathological analysis.

### Cell culture

Human umbilical vein cells were obtained from Lonza (pulled version) and maintained in EGM-2 media (Lonza). Tumor cells were grown in Dulbecco’s Modified Eagles Medium (DMEM) supplemented with 10% Fetal Bovine Serum (FBS) and 100 U/ml penicillin, 100 μg/ml streptomycin. Cells were cultured at 37 °C in 5% CO_2_. Tumor cell authentication (STAB VIDA) and mycoplasma testing (Eurofins) were performed previously to the experiments.

### Microscopy

#### Immunofluorescence

For the quantification of blood vessels in tumors, 6–10 μm thick slices from frozen tumor samples were stained using an anti-CD31 antibody (553370, BD) according to standard protocols. The number of CD31+ blood vessel was quantified manually 5 ROI with the highest number of blood vessels (hot spots) per tumor using a Zeiss LSM710 confocal microscope and 20× magnification.

#### Bodipy staining

Cells were fixed with 4% paraformaldehyde in PBS for 15 min, washed 3 times, and then stained with 0.1 μg/ml Bodipy (Invitrogen) in PBS, at RT for 60 min. Cells were then washed three times with PBS and mounted with Vectashield mounting medium containing DAPI (Vector Laboratories). Images were captured using a Zeiss LSM710 confocal microscope.

#### Transendothelial migration assay on transwells

For the transendothelial migration assay on transwells, 0.4 µm pore size inserts were used to allow cancer cell directed migration towards a chemoattractant and avoid endothelial cell migration (which was observed in inserts with bigger pore sizes). Tumor cell transendothelial migration was then assessed by the quantification of cancer cells that crossed the endothelial monolayer as visualized by confocal microscopy—Z stack images—of VE-cadherin and GFP stained samples. In detail on day 1, 0.5 × 10^5^ endothelial cells (HUVECs) were plated in EGM-2 on top of 6.5 mm diameter inserts with 0.4 µm pore size (Costar). Bottom chambers were filled with 600 µl of EBM-2 without FBS. The next day both top and bottom media were replaced and cells kept in the culture in order for the monolayer to mature in terms of junction formation. On day 3 media on endothelial cells was replaced by EBM-2 without FBS at the top chamber and EBM-2 without FBS and supplemented with 10 ng/ml HGF and 1 × 10^4^ MDA-231 GFP+ cells either untreated or previously treated with LDL for 6 h were added on the top of the endothelial monolayer. Six hours later cells were fixed with 4% PFA. Filters were then cut from the insert for staining with anti-VE-cadherin and anti-GFP antibodies for immunofluorescence and confocal imaging using a Zeiss LSM 710 microscope. The percentage of transmigrated cells was calculated by quantifying the number of cells that crossed the endothelial monolayer over the total number of cells in each region of interest (ROI). Around 50 tumor cells were quantified for both control and LDL conditions from 5 ROI.

#### Time-lapse

For images in 2D, 1 × 10^5^ tumor cells were plated on 12 well plates and the day after treated with either 100 μg/ml LDL or the same volume of control (150 mM NaCl, 0.01% EDTA) for 6 h. After that, cells were trypsinized and 0.5 × 10^4^ cells were plated on top of confluent monolayers of HUVECs, that had been plated the day before at 2 × 10^4^ cells per well on top of 0.2% gelatin (8 well 1 cm^2^ area ibidi plates). Cells were imaged on a Zeiss Axio Observer under controlled CO_2_ and temperature. Pictures were taken every 5 min at both transmitted (green channel) and reflected light using a Zeiss Axio Observer under controlled CO2 and temperature.

#### 3D branch formation assay

The 3D branch formation assay was performed on 8 well 1 cm^2^ area plates (Ibidi), that were previously coated with 200 µl of Matrigel (1 h before the addition of the cells). Cells were pre-treated for 6 h with either 100 µg/ml LDL or the same volume of control solution (150 mM NaCl, 0.01% EDTA) and then plated on top of Matrigel in the presence of either 100 µg/ml LDL or the same volume of control solution. Cells were plated at 2.5 × 10^4^ HUVECs for HUVECS only; or 2 × 10^4^ HUVECs + 0.5 × 10^4^ tumor cell in the co-cultures. 16 h later cells were imaged on a Zeiss Cell Observer under controlled CO_2_ and temperature. Pictures were taken at both transmitted and reflected light. The number of bifurcations and the percentage of branches containing intercalated cancer cells from 6 ROI were quantified manually.

#### Cholesterol quantification

Total cholesterol quantification was performed on cell lysates using the Amplex^®^ Red Cholesterol Assay Kit (Invitrogen). Cells were first placed on ice, washed with ice-cold PBS 2 times, and then lysed with Amplex^®^ Red reaction buffer 1× (Invitrogen). Lysates were then moved to a cold Eppendorf on ice and centrifuged at 12,000 rpm for 20 min at 4 °C. Subsequent steps were performed according to the manufacturer’s indications.

#### Western blotting

For protein extraction, cells were lysed with RIPA buffer (Merck) supplemented with cOmplete™ Protease Inhibitor Cocktail (Roche) and the solution was kept for 10 min on ice. Lysates were then centrifuged at 12,000 rpm for 20 min. 5 µl of each supernatant was collected for protein concentration determination using the DC protein assay (Biorad) and the remaining collected and mixed with 4× Laemmli buffer (Bio-Rad) supplemented with 2% dithiothreitol (Bio-Rad) in a 1:4 proportion. The resulting solutions were heated at 95 °C for 5 min and stored at − 20 °C until they were needed for subsequent steps. Proteins were separated using 10% SDS-PAGE gels and transferred to nitrocellulose membranes. The membranes were then blocked with 5% milk solution and incubated with the primary antibody (anti-SERPINE2, ab154591, Abcam^44^), the secondary antibody donkey anti-goat horseradish peroxidase-labeled (Santa Cruz Biotechnology (1:5000 in 5% milk solution), for 1 h at room temperature, followed by three 10 min washes with TBST. To detect the β-actin as a loading control, the membranes were incubated with mouse anti-β-actin (Merck) (1:2000 in TBST) for 1 h and, after three 10 min washes with TBST, were also incubated with the secondary antibody Goat anti-mouse horseradish peroxidase-labeled (Promega) (1:5000 in 5% milk solution) for 1 h. Development was performed using ECL prime and ECL start western blotting detection reagents (GE Healthcare) for the detection of SERPINE2 and Actin, respectively and chemiluminescence detected on a GE Healthcare Amersham 680 CCD imager. Band quantification was performed using Fiji software and SERPINE2 band intensity was corrected for Actin band intensity of each sample. Reversion experiments on LDL induction of SERPINE2 expression were performed using 20 µg/ml anti-LDLR and 50 µg/ml of Nystatin (Sigma).

### Statistical analysis

Statistical analysis were performed using GraphPad Prism 8.3 software. To test for a relationship between the presence of CTCs in mice and the diet with 95% confidence interval, a contingency analysis and a Fisher’s exact test to compute the p-value were used. In the remaining analysis, data are presented as mean ± standard deviation (SDEV) and a Student’s *t*-test except for Fig. 3C when an ANOVA was used to assess differences between the experimental groups. Differences were considered significant when p < 0.05 (*).

### Supplementary Information

Supplementary Video 1.

Supplementary Video 2.

Supplementary Video 3.

Supplementary Figures.

## Data Availability

Data presented in this work is contained within figures, including supplementary and any clarification will be given upon request, please contact anamagalhaes@fm.ul.pt or sergiodias@medicina.ulisboa.pt for that matter.
