# Supplementary material for: A high-cholesterol diet promotes the intravasation of breast tumor cells through an LDL–LDLR axis
Source: Sci Rep. 2024 Apr 24;14:9471. doi: 10.1038/s41598-024-59845-3 (PMC11043359; doi:10.1038/s41598-024-59845-3)
Supplement: Supplementary file 4 — Supplementary Figures. [file 41598_2024_59845_MOESM4_ESM.pdf]

Supplementary Figure 1

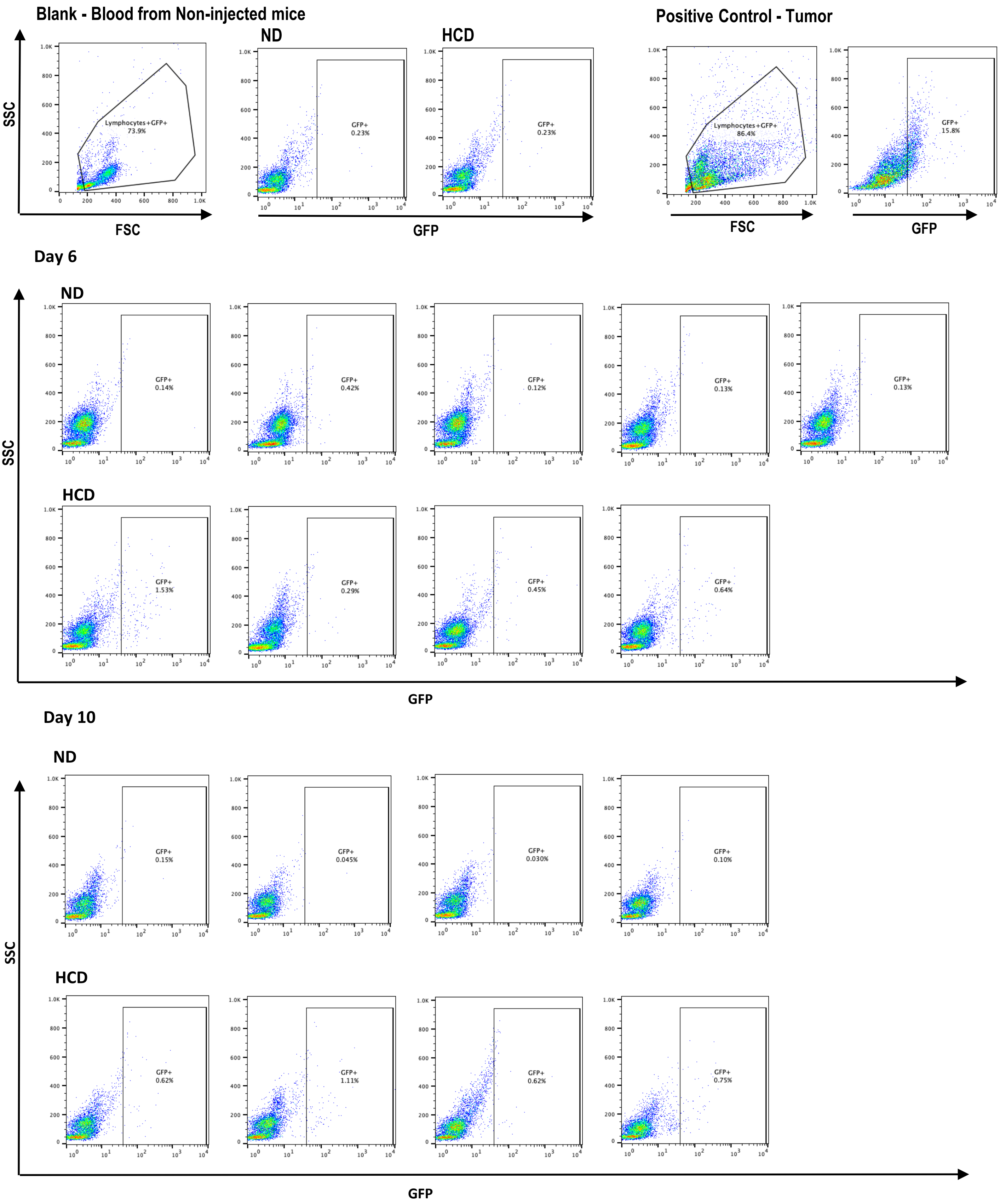

Supp. Fig1: **Raw data for Fig 1A and B.** FACs analysis of blood samples collected from BALB/c mice that were fed with either a normal diet (ND) or high cholesterol diet (HCD) for two weeks and then injected (or not) with 4T1 GFP-expressing breast tumor cells at the mammary fat pad. Six and ten days after injection, blood samples were collected and analyzed by flow cytometry (FC) to search for the presence of GFP+ cells. Blood collected from mice from each experimental group that were not injected with GFP+ tumor cells were used as controls for each experiment (blank – blood from non-injected mice).

Supplementary Figure 2

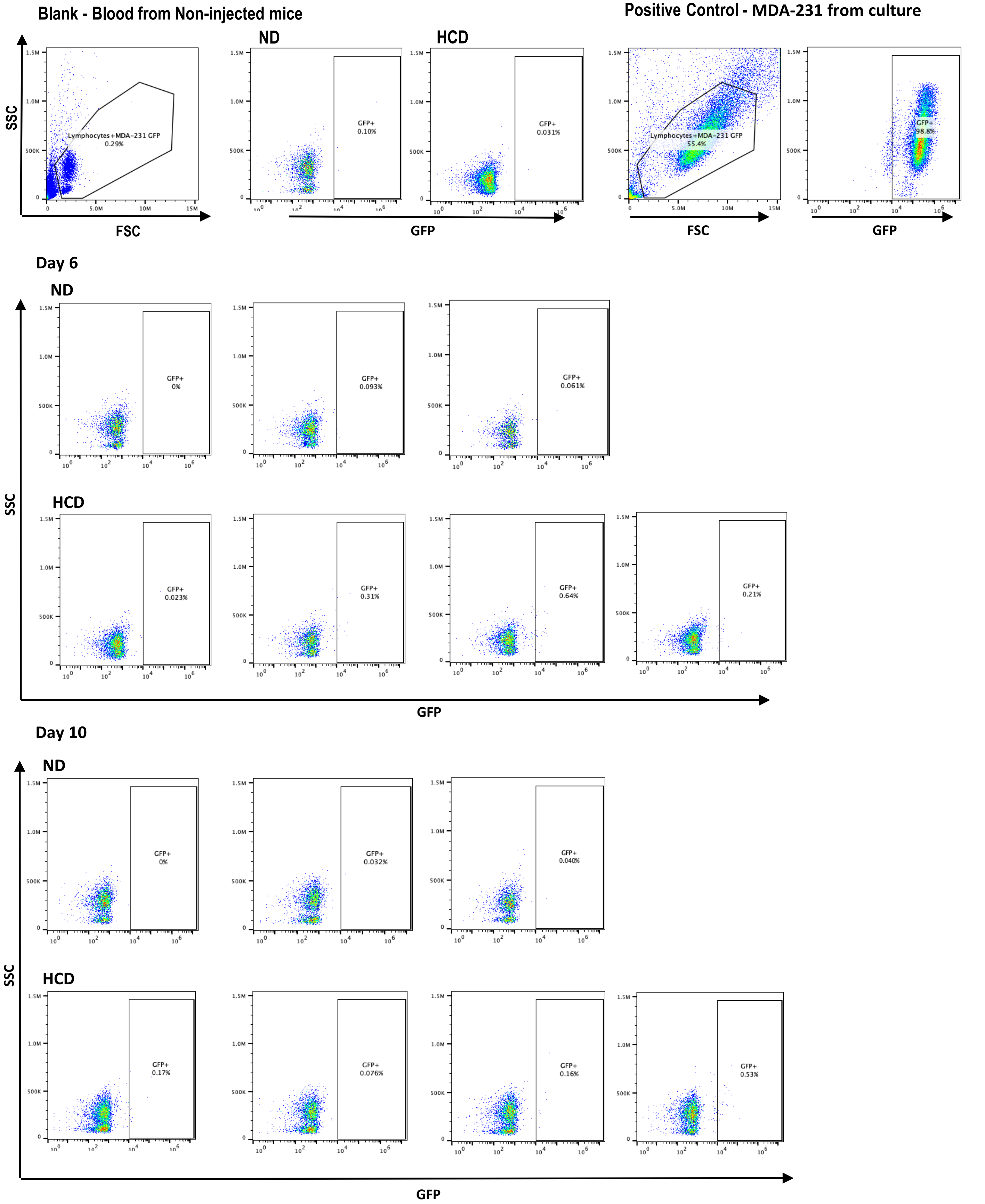

Supp Fig2: Raw data for Fig 1E. FACs analysis of blood samples collected from NSG mice that were fed with either a normal diet (ND) or high cholesterol diet (HCD) for two weeks and then injected (or not) with MDA-231 GFP-expressing breast tumor cells at the mammary fat pad. Six and ten days after injection, blood samples were collected and analyzed by flow cytometry (FC) to search for the presence of GFP+ cells. Blood collected from mice were not injected with GFP+ tumor cells were used as controls for each experiment (blank – blood from non-injected mice).

Supplementary Figure 3

A

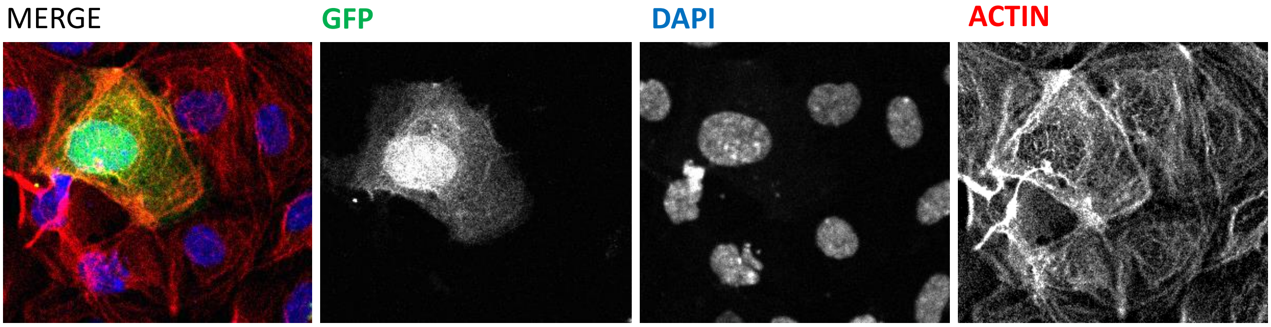

B

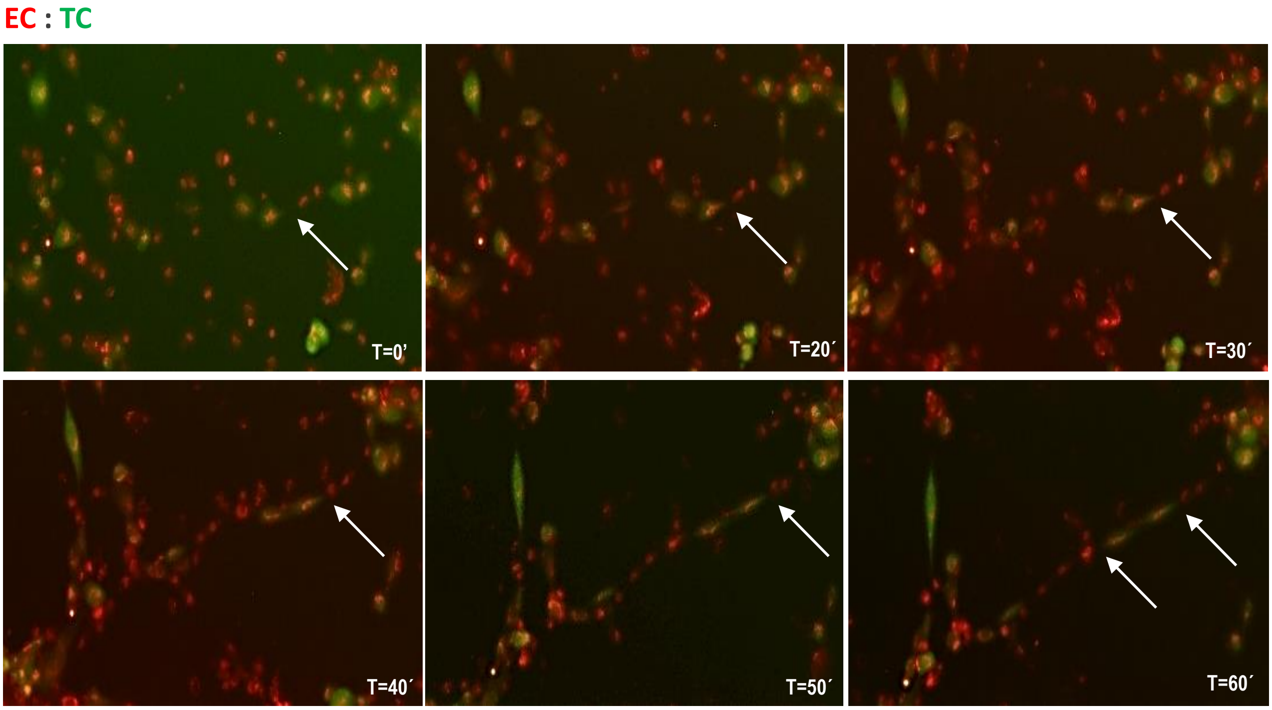

Supp Fig3: Tumor cell intercalation with endothelial cells A) Confocal microscopy showing a tumor cell (GFP+) intercalated with endothelial cells (non-GFP). **B)** Still images from movie 3 - a time lapse imaging of cancer cells GFP+ (Green) and endothelial cells stained with a vital dye (cell tracker orange, invitrogen) co-plated on top of matrigel. Arrow points for an intercalation event: a tumor cell contributing to form a branch by intercalating between endothelial cells.

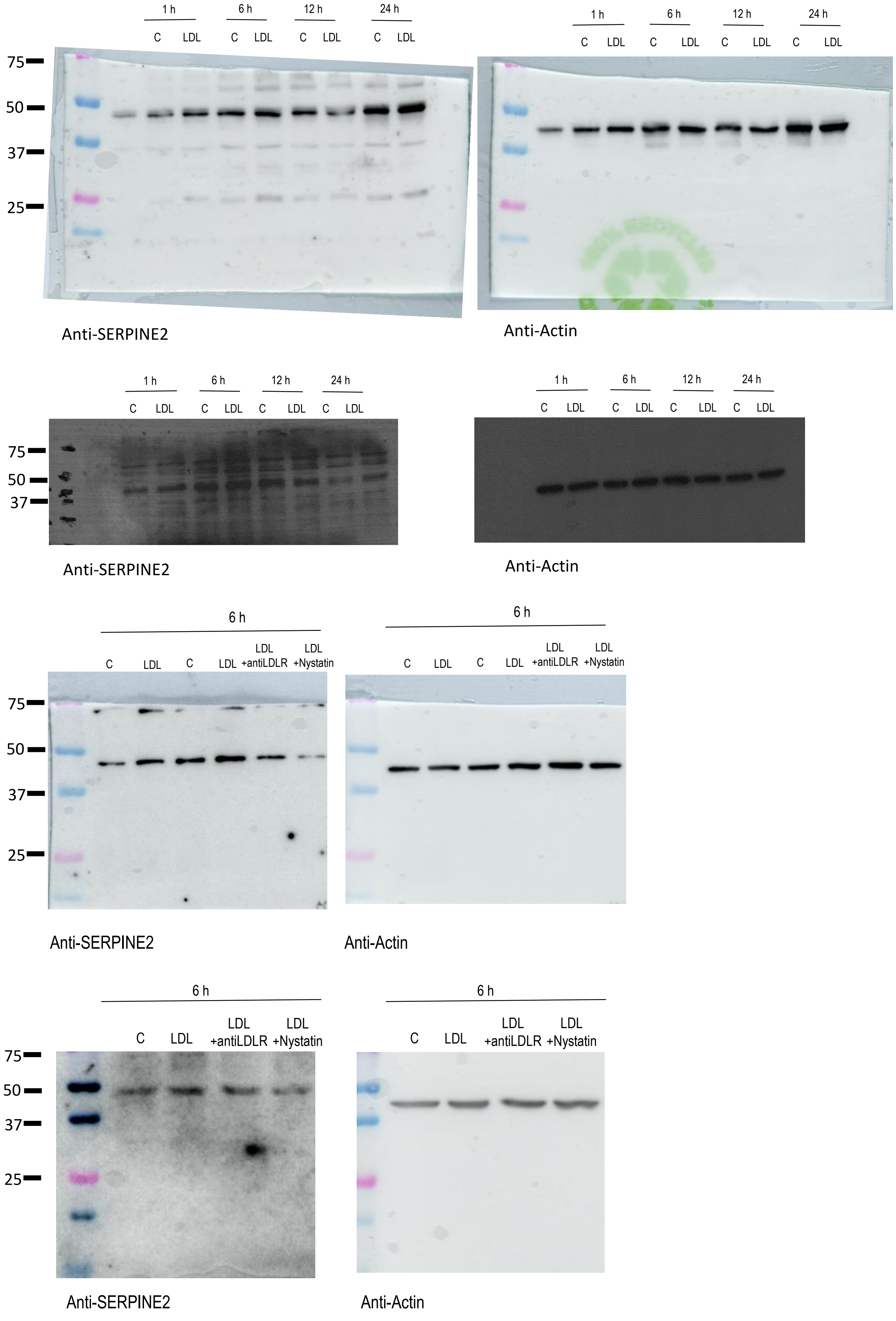

Supp Fig4: Full western blots of cropped images in manuscript´s figure2.

Supplementary Figure 5 – I

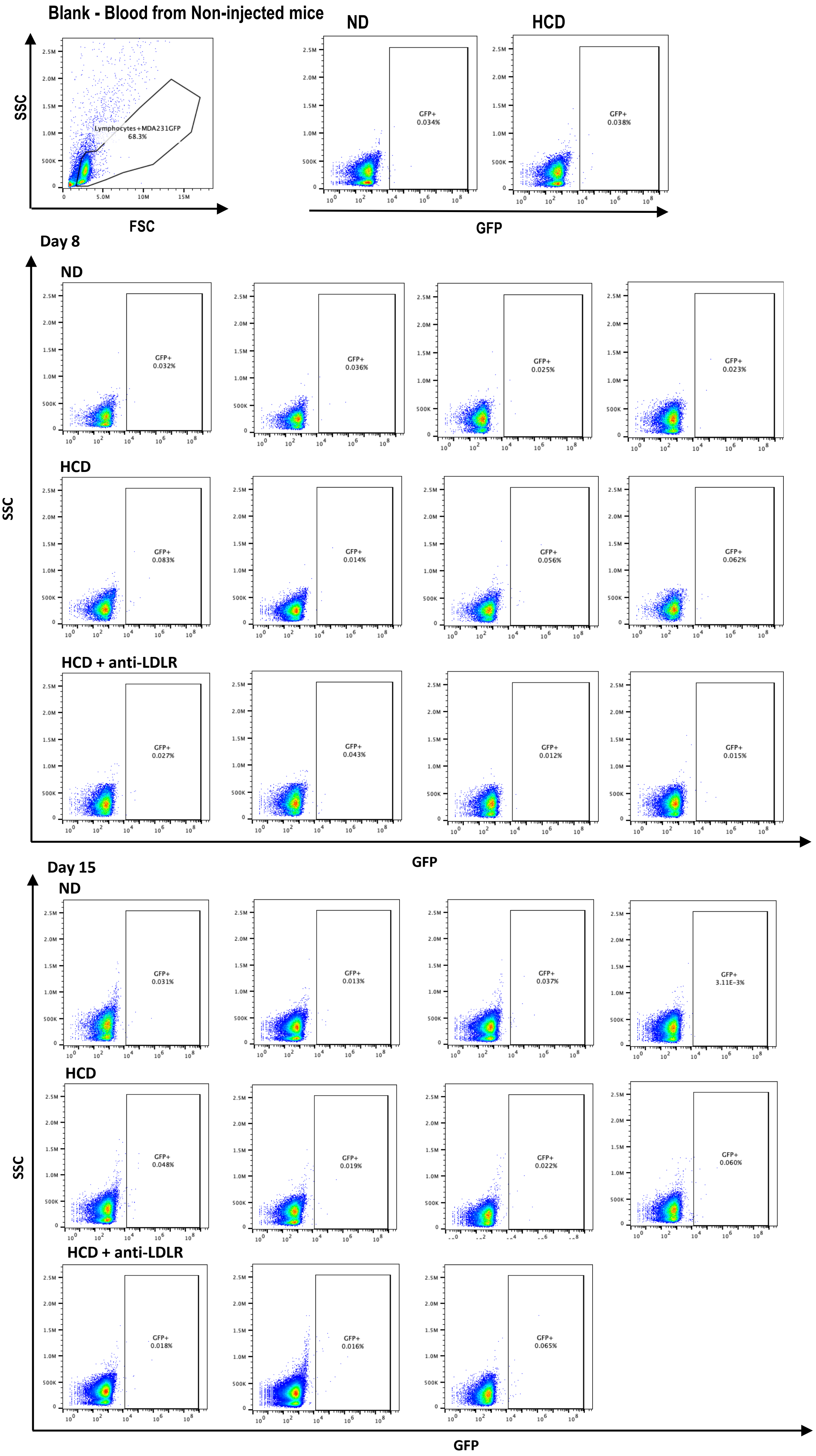

Supplementary Figure 5 – II

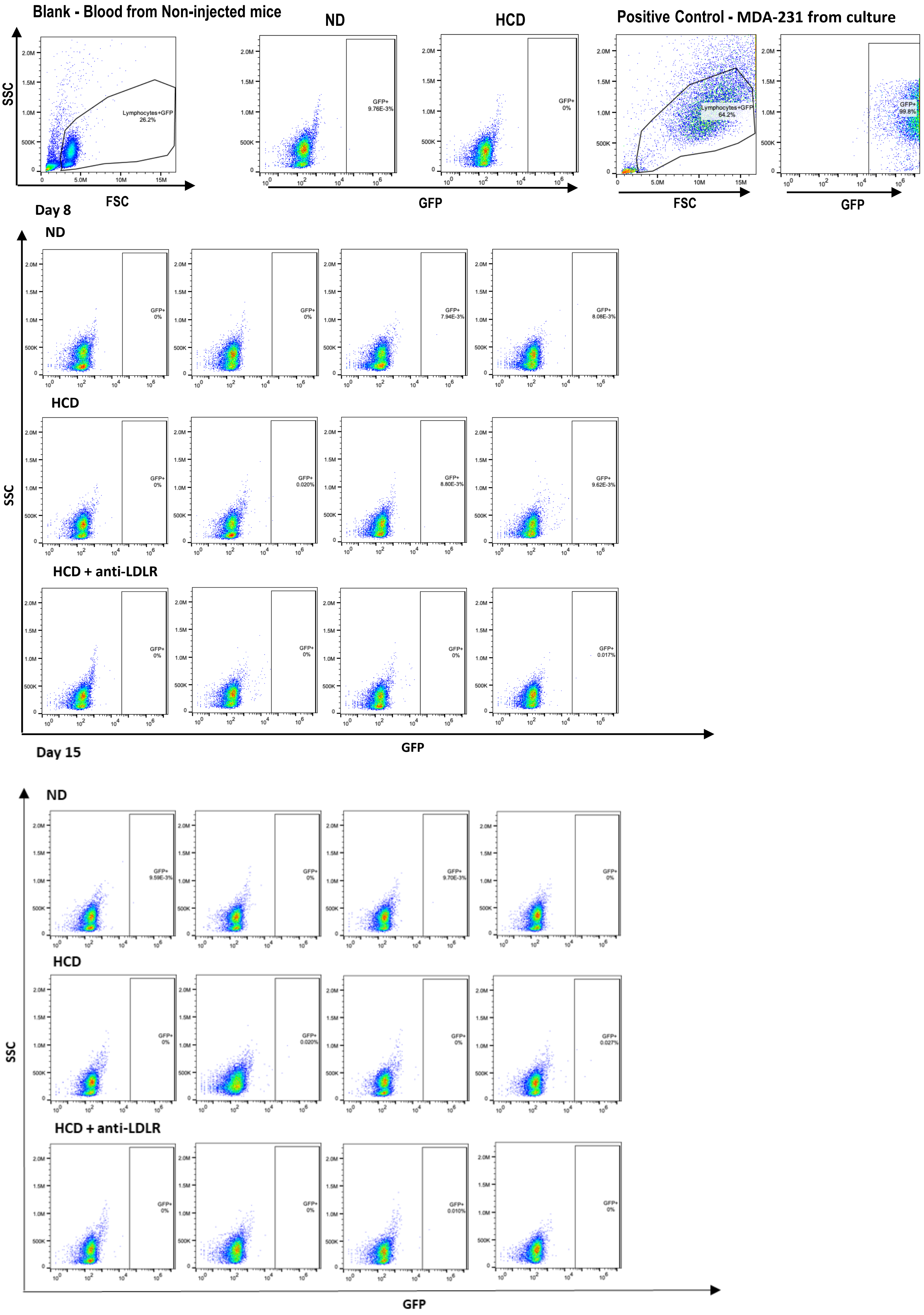

Supplementary Figure 5 – III

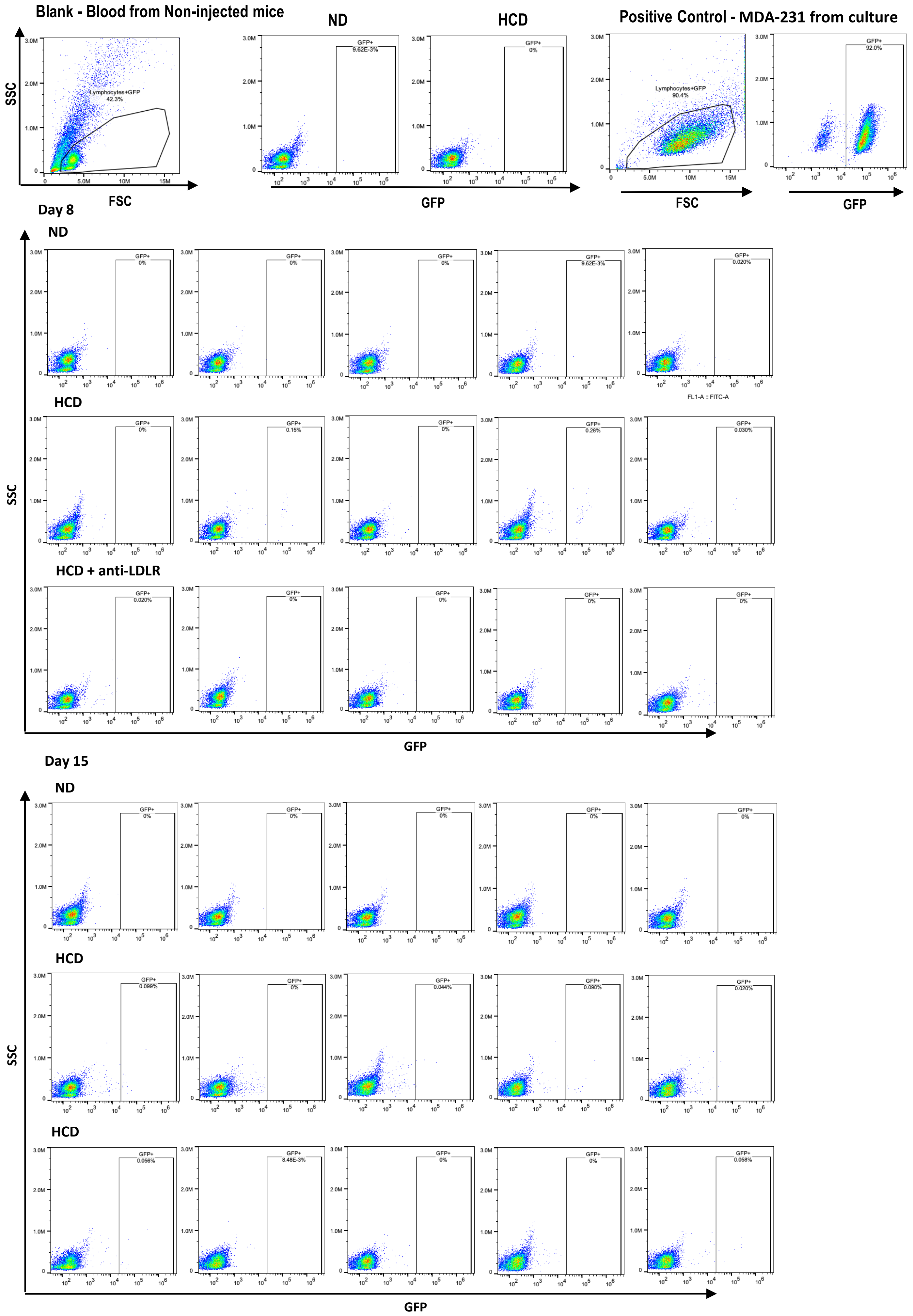

Supplementary Figure 5 – IV

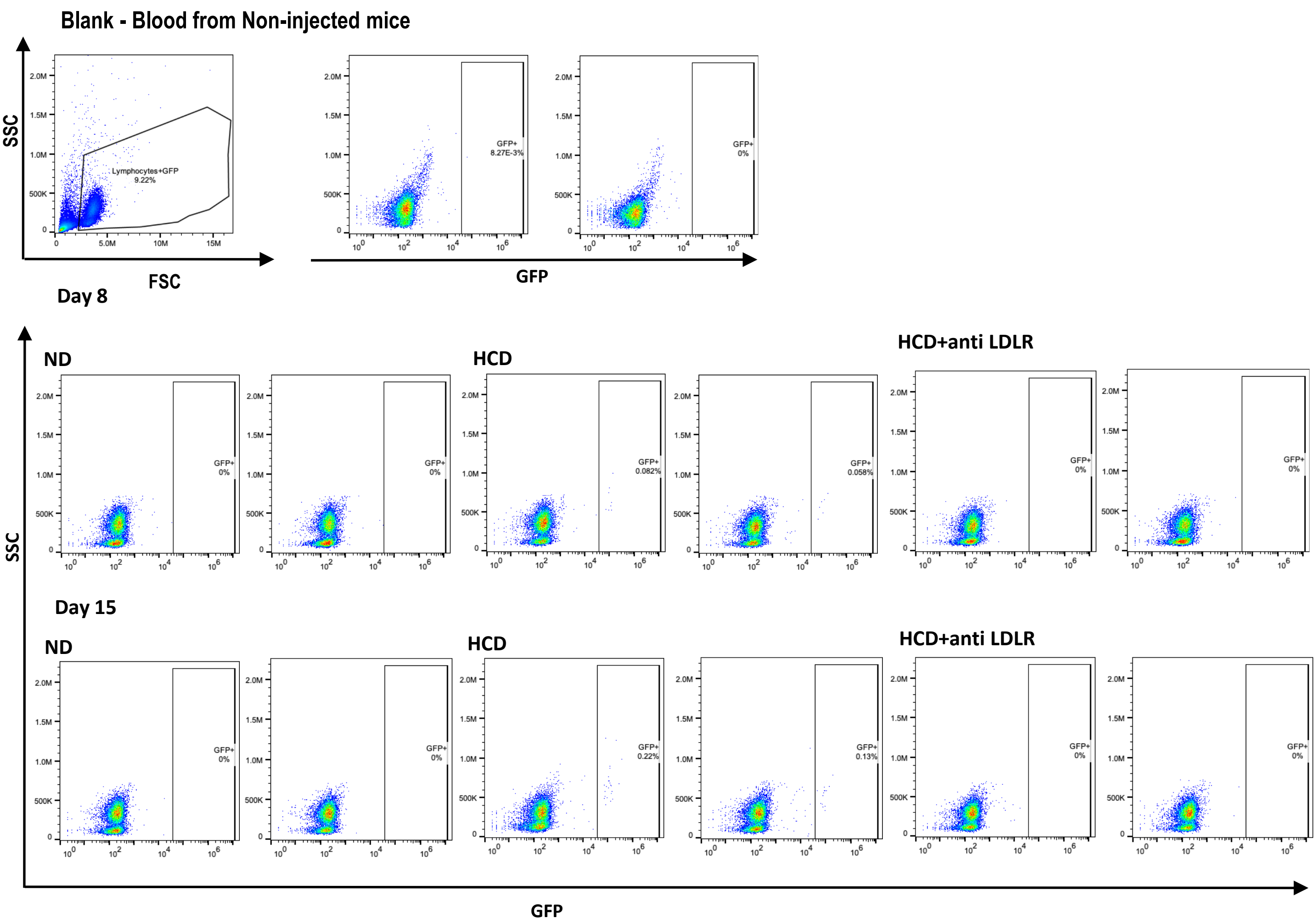

Supp Fig5: Raw data for Fig 3 B (CTCs from mice fed on ND or HCD). Figure is divided in I, II, III and IV corresponding to four independent experiments**.**

Supplementary Figure 6 - I

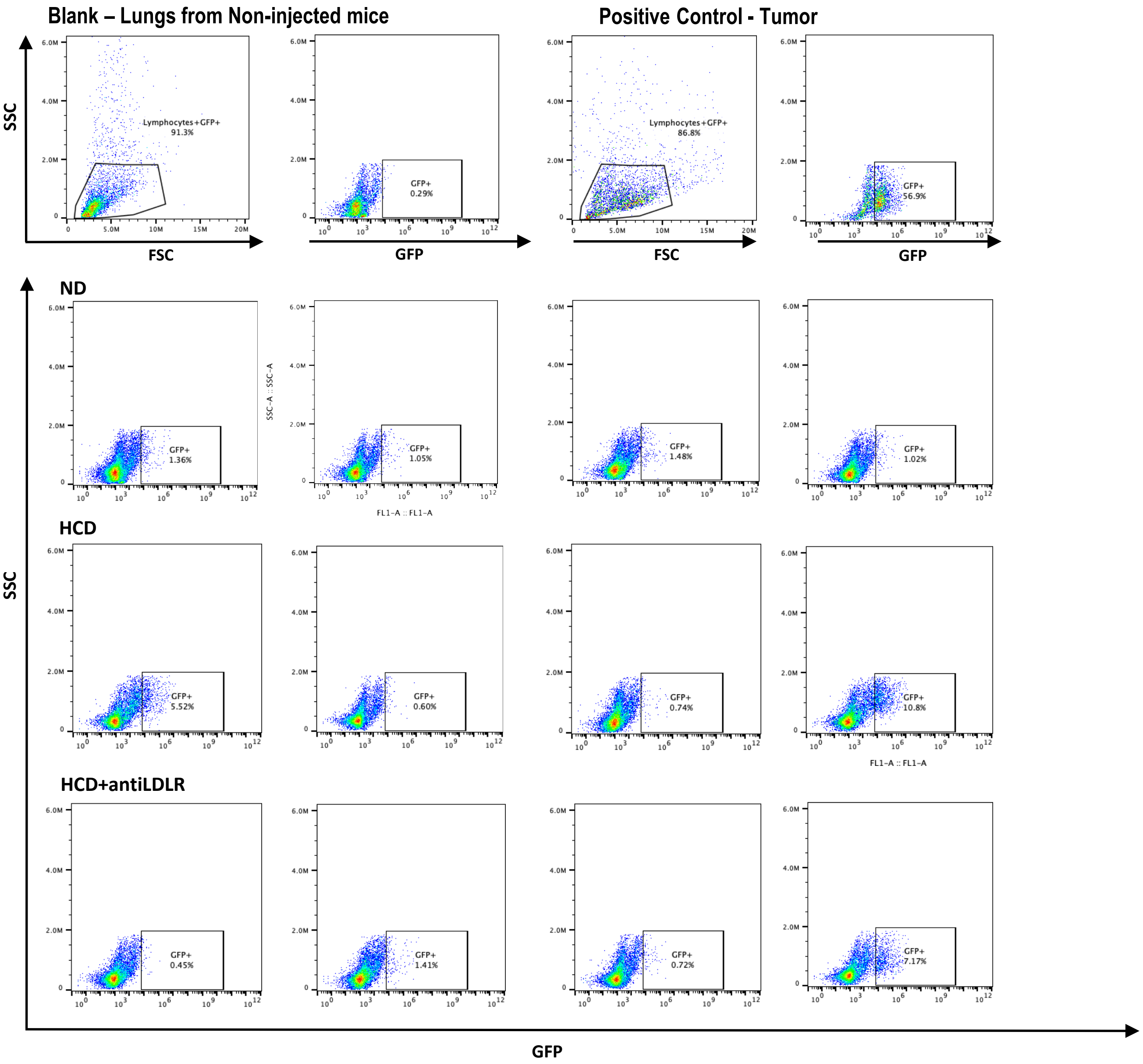

Supp Fig6: Raw data for Fig 3 C (disseminated tumor cells in mice fed on ND or HCD). Figure is divided in I, II, III and IV, corresponding to four independent experiments**.** (6-II, 6-III and 6 IV below).

Supplementary Figure 6 - II

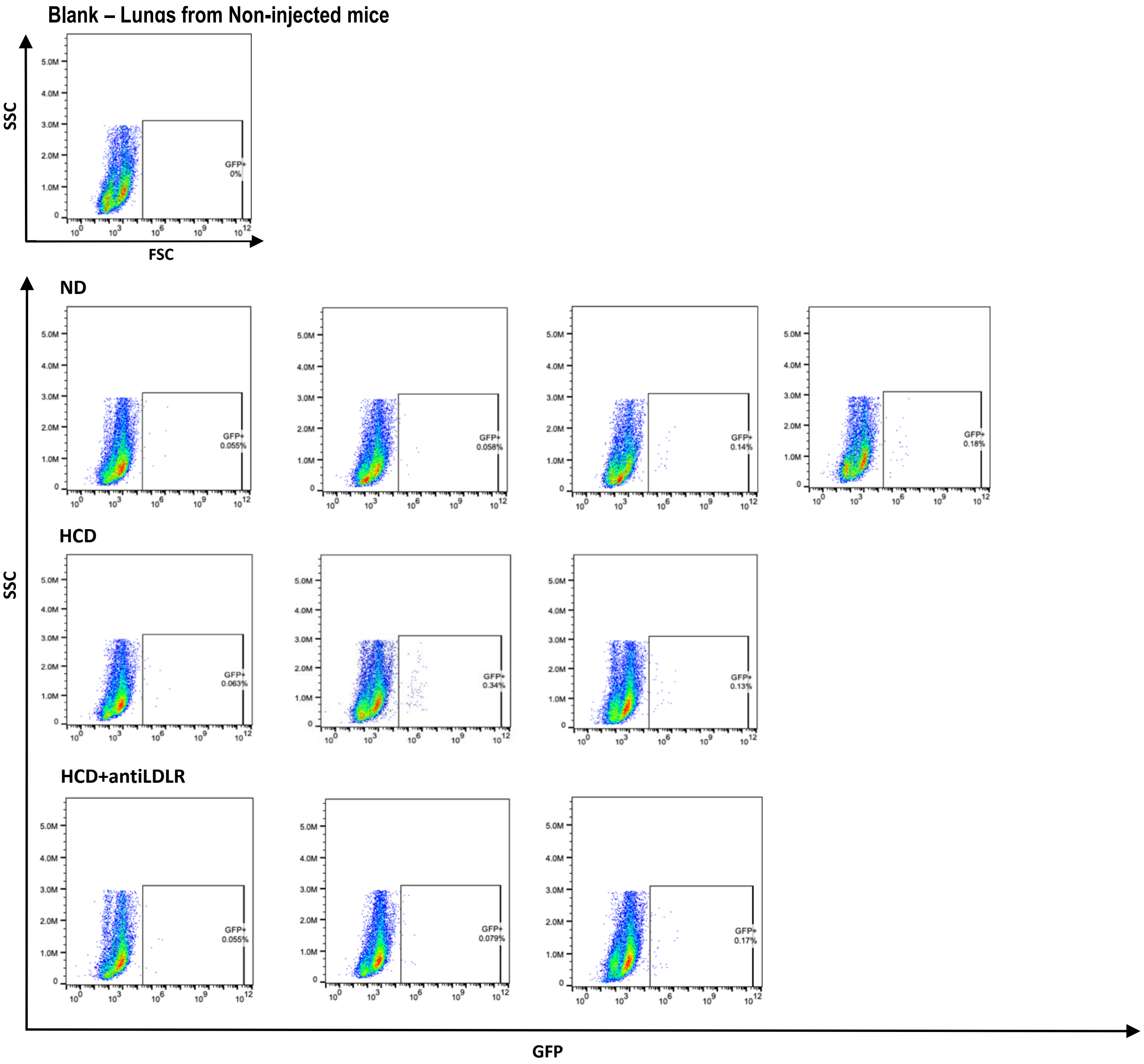

Supplementary Figure 6 - IV

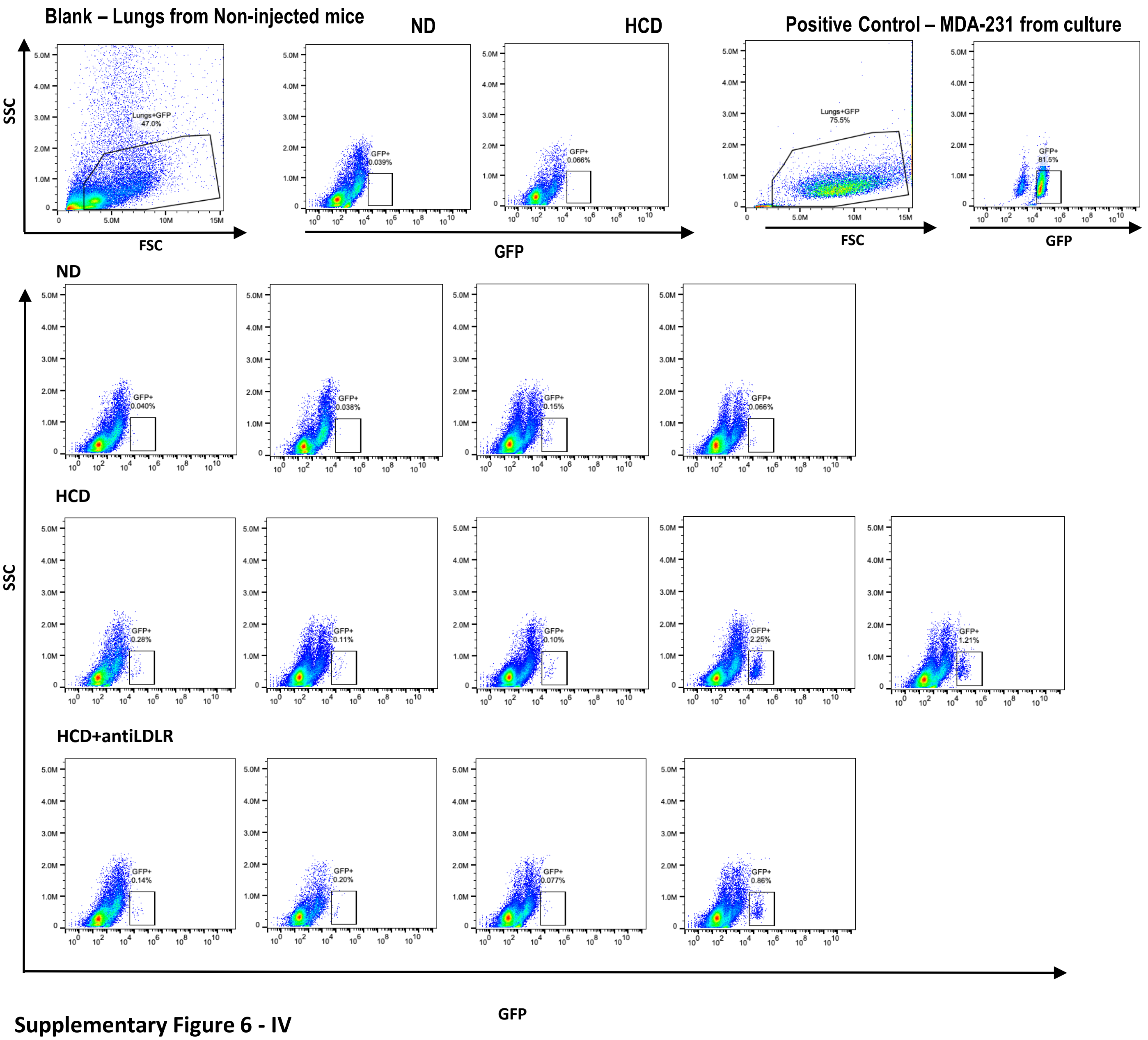

Supplementary Figure 6 - IV

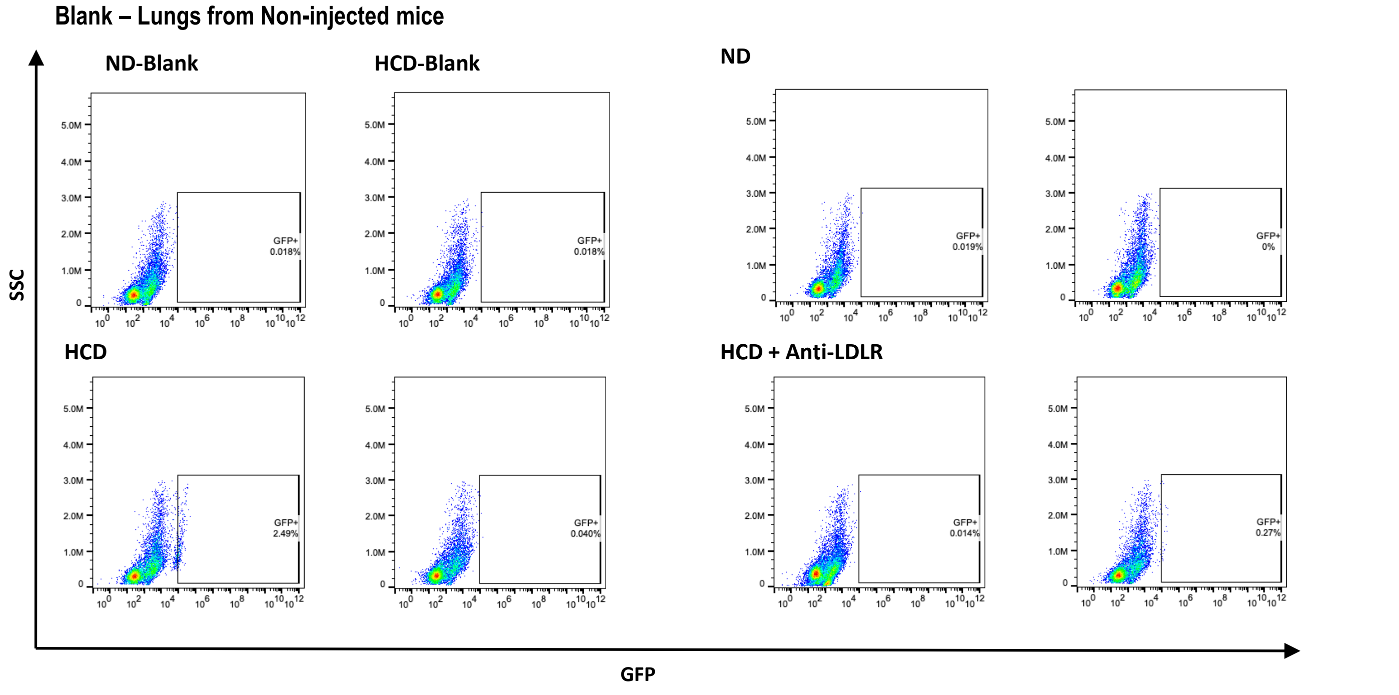

Supplementary Figure 7

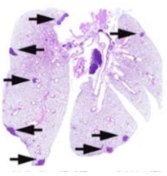

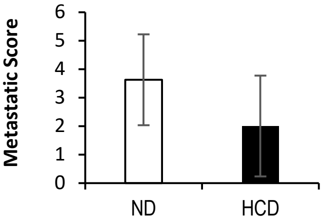

# metastatic foci	score
0	0
1 to 5	1
5 to 10	2
10 to 20	3

cells per foci	score
0	0
up to 10	1
10 to 20	2
> 20	3

Supp Fig7: Tail-vein injection of MDA-231 cells in Balb-SCID mice. Mice were separated in two groups and fed either on normal diet (ND) or on HCD for 2 weeks. MDA-231 cells were injected and the tail vein of each mice and 2 weeks later mice were sacrificed, lungs collected and assessed for the presence of metastasis by the histopathology unit. A metastatic score was used to quantify the extent of metastatic outgrowth in each mouse.
